# Limited physical literacy outcomes in Singapore children: Implications for public health practice

**DOI:** 10.1016/j.jesf.2026.200517

**Published:** 2026-09-02

**Authors:** Vanes Ling Li Tay, Shawnda A. Morrison, Natarajan Padmapriya, Jonathan Y. Bernard, Falk Müller-Riemenschneider, Yung Seng Lee, Yap Seng Chong, Fabian Yap, Johan Gunnar Eriksson, Jason Kai Wei Lee

**Affiliations:** a Human Potential Translational Research Programme, Yong Loo Lin School of Medicine, National University of Singapore, Singapore, 117597, Singapore; b Saw Swee Hock School of Public Health, National University of Singapore, Singapore, 117549, Singapore; c Department of Obstetrics & Gynaecology, Yong Loo Lin School of Medicine, National University of Singapore, 119228, Singapore; d Centre for Research in Epidemiology and StatisticS (CRESS), Université Paris Cité and Université Sorbonne Paris Nord, Inserm, INRAE, Paris, F-75004, France; e Desbrest Institute of Epidemiology and Public Health (IDESP), Université de Montpellier, Inserm, Montpellier, F-34090, France; f Institute for Human Development and Potential (IHDP), Agency for Science, Technology and Research (A*STAR), Singapore, 117609, Singapore; g Department of Paediatrics, Yong Loo Lin School of Medicine, National University of Singapore, Singapore, 119228, Singapore; h Khoo Teck Puat-National University Children's Medical Institute, National University Health System, Singapore, 119074, Singapore; i Division of Medicine, KK Women's and Children's Hospital, Singapore, 229899, Singapore; j Duke-NUS Medical School, National University of Singapore, Singapore, 169857, Singapore; k Lee Kong Chian School of Medicine, Nanyang Technological University, Singapore, 636921, Singapore; l Department of General Practice and Primary Health Care, University of Helsinki, Helsinki, 00290, Finland; m Department of Physiology, Yong Loo Lin School of Medicine, National University of Singapore, 117593, Singapore; n Heat Resilience & Performance Centre, Yong Loo Lin School of Medicine, National University of Singapore, 117510, Singapore

**Keywords:** GUSTO, Health-related fitness, Physical activity, Socio-economic factors, Southeast Asia

## Abstract

**Background/objectives:**

Adequate physical literacy (PL) in children is a potential preventive measure against non-communicable diseases. Direct measurement of PL is often absent in population-wide studies, particularly in East and Southeast Asia. We assessed PL and associated individual- and environmental-level factors in Singapore children.

**Methods:**

726 participants (373 boys, 353 girls; age: 11.0 ± 0.2 years) from a birth cohort study (GUSTO) completed a self-administered questionnaire in the Canadian Assessment of Physical Literacy Second Edition (CAPL-2) to assess affective and cognitive domains of PL. A subset of 360 children (187 boys, 173 girls) completed a modified fitness test-battery to assess physical competence. Chi-square tests of independence compared sex and ethnicity differences in PL scores. A modified three-domain composite Overall PL score was derived. Pearson's correlation assessed PL scores against self-reported moderate-to-vigorous physical activity (MVPA) duration. Regression analysis examined associations of socio-economic status (SES) with PL, adjusting for sex, ethnicity, and MVPA.

**Results:**

72.8% of children were classified within the lower category bins of the modified Overall PL scoring framework. Self-reported MVPA duration was positively associated with modified Overall PL score (r(358) = 0.23, *p* = 0.01). After adjustment, the regression model revealed that household income was not associated with modified Overall PL scores (all *p* > 0.05), while higher maternal education remained associated with higher scores (all *p* < 0.01).

**Conclusion:**

Low levels of PL observed in Singapore school children warrant concern for public health and disease prevention. These findings, alongside similarities in educational systems, cultural influences, and socioeconomic factors shared with countries within the region, highlight an urgent need to prioritize physical activity and PL in children across Asian contexts.

## 1 Introduction

Engaging in consistent regular physical activity (PA) can reduce morbidity and mortality.^1^, ^2^, ^3^ According to World Health Organization (WHO), children and adolescents aged 5 to 17 should complete an average of at least 60 min of daily moderate-to-vigorous physical activity (MVPA) per week to achieve meaningful health benefits.^4^^,^^5^ Individuals who maintain adequate levels of MVPA during childhood are more likely to accrue adequate PA for disease prevention as they age.^6^, ^7^, ^8^ Despite these well-established health benefits, data from 57 countries have shown that only an estimated 27-33% of children and adolescents aged 5-17 years old meet PA guidelines, according to the Active Healthy Kids Global Alliance Global Matrix 4.0 Report.^9^ High-income Asia Pacific countries, and East and Southeast Asian countries formed two of the top three regions which reported the highest percentages of school-going children who are insufficiently active, at approximately 92% and 86%, respectively.^10^

The concept of *physical literacy* (PL) has emerged as a foundational model to assist in facilitating and maintaining lifelong PA, fitness, and disease prevention.^11^ PL is defined as “the motivation, confidence, physical competence, knowledge and understanding to value and take responsibility for maintaining purposeful physical pursuits/activities throughout the lifecourse”.^12^ Physically-literate individuals are more likely to utilize information from their social and physical environment to make better movement decisions and transfer their skills to a range of challenging movement situations in a creative, confident, and enjoyable manner.^12^^,^^13^ Accordingly, they can sustain healthy PA habits and achieve long-term positive health outcomes,^14^^,^^15^ with individual- (e.g., sex, ethnicity) and environmental contextual- (e.g., cultural expectations, weather conditions) level factors moderating this reciprocal relationship between PL and PA.^16^

Specific to Asia, positive correlations have been found between perceived and actual PL in primary school children in Hong Kong,^17^ as well as between perceived PL and self-reported PA in adolescents.^18^ Extending this line of enquiry, Li et al. (2021) tested the directionality of these relationships and reported a reverse pathway, whereby PA participation predicted actual PL, rather than the reverse.^19^ These studies underline a complex, multi-directional relationship between PL and PA within Asian-specific contexts. For example, one study comparing PL between Chinese and Greek children through the use of a multi-component tool, reported Greek children faring better than Chinese children, (although notably, children from both regions generally demonstrated inadequate levels of PL).^20^ Specifically in Southeast Asia, a preliminary validation of the Canadian Assessment of Physical Literacy second edition (CAPL-2) in Indonesian children aged 8 to 12 years observed low PL levels, suggesting that inadequate PL may be a concern across the greater Southeast Asian region.^21^ Similarly, a multi-component PL tool was adapted to compare differences in physical competence in Thai children from rural and urban school areas.^22^ Children in rural schools demonstrated greater overall physical competence, suggesting facilitative environments may impact greater physical development.

To date, no study has examined PL of Singapore children. Singapore is a highly diverse, multi-ethnic population, comprising of Chinese (74%), Malays (14%), Indians (9%), and other minority ethnic groups (3%),^23^ reflecting demographic features that are shared with several jurisdictions across the East and Southeast Asian region. Previously, sex, ethnicity, and socioeconomic status (SES) have been identified as key sociodemographic determinants of children's movement behaviors. For example, girls of Malay ethnicity, and lower SES, demonstrated the greatest decline in MVPA across childhood in Singapore.^24^ Considering East and Southeast Asian regions account for the largest proportion of global population (29%),^25^ it is of paramount public health concern to accurately assess and quantify PL levels of children, in order to contribute accurate regional evidence and better customize specific PA interventions to improve PA behavior. Although Singapore's unique status as a high-income,^26^ hyper-urbanized city-state^27^ may limit direct generalizability of any findings to the broader region,^28^ its multi-ethnic population,^23^ academically competitive culture,^29^ and tropical climate do share critical environmental features common to many East and Southeast Asian jurisdictions,^30^, ^31^, ^32^ making Singapore a contextually-relevant case study.

In this study, we sought to determine PL levels of Singapore children for the first time, to identify which specific domains showed the greatest potential for public health initiatives aimed at improving PL in this population. We examined key individual- and environmental contextual-level factors (i.e., sex, ethnicity, SES predictors), which could influence PL outcomes in Singapore children. We hypothesized that: (i) Singapore children would exhibit low PL levels, consistent with the pattern of low PL reported across other Asian populations, (ii) boys, and children with higher SES, would demonstrate higher Overall PL scores, and (iii) self-reported MVPA levels would be associated with PL outcomes.

## 2 Methods

### 2.1 Study design and participants

Study participants were part of the prospective “Growing Up in Singapore Towards healthy Outcomes (GUSTO)” birth cohort study. The participating mothers were included in the cohort study if they met the following criteria: (i) pregnant women aged ≥18 years; (ii) attended a first trimester antenatal dating ultrasound scan at National University Hospital (NUH) or KK Women's and Children's Hospital (KKH) between June 2009 and September 2010; (iii) Singapore citizens or permanent residents of Chinese, Malay, or Indian ethnicity with homogeneous parental ethnic background; (iv) intended to deliver at NUH or KKH and reside in Singapore for the subsequent 5 years; and (v) agreed to donate birth tissues (cord, placenta, and cord blood) at delivery.^33^ They were excluded if they had any of the following: (i) receiving chemotherapy; (ii) use of psychotropic drugs; or (iii) diagnosis of type I diabetes mellitus.^33^ More details on the study protocol are available elsewhere.^33^ No additional child-specific inclusion or exclusion criteria were imposed for the present study. Although physical disability status was not formally assessed (or used as an eligibility criterion), no children with apparent disabilities participated in the study. This study received ethical approval from the National Healthcare Group Domain Specific Review Board (D/09/021, 2019/2655/E) and the SingHealth Centralised Institutional Review Board (2009/280/D, 2019/2655/E). Informed consent was provided by all participating mothers and/or fathers, and children provided assent for all assessments once reaching 6 years of age. Data for the present study was collected during the follow-up at age 11 years.

### 2.2 Assessment of the affective and cognitive domains

During the children's clinical visits, the Motivation and Confidence domain and the Knowledge and Understanding domain were assessed using the self-reported questionnaire from the CAPL-2 manual.^34^ The CAPL-2 is one of three available multi-component tools used to assess PL as an entity,^34^^,^^35^ and has been applied and validated in other populations.^21^^,^^36^^,^^37^ Specifically within Asia, the questionnaire components of the CAPL-2 have been validated, and translated for use in Chinese children aged 8-12 years.^36^ Preliminary construct and content validity has been established for Indonesian children within the same age range.^21^ Collectively, this evidence supports the applicability of the CAPL-2 framework beyond the Canadian context. A total of 726 children completed the questionnaire in full. The Motivation and Confidence domain assessment consists of 12 questions which delve into four components: predilection, adequacy, perceived competence satisfaction and intrinsic motivation.^34^ This domain was scored out of 30 points. The Knowledge and Understanding domain consists of 10 questions targeting four aspects: daily PA recommendations, aerobic endurance terminology, muscular endurance terminology, and techniques for improving physical competence.^34^ This domain was scored out of 10 points.

The Motivation and Confidence score and the Knowledge and Understanding score of each child was used to assign them to one of four possible interpretive categories (i.e., Beginning, Progressing, Achieving, and Excelling) for each domain, based on age- and sex-specific score percentiles referencing data from 10,000 Canadian children.^34^ The distribution of children across the different interpretive categories for each domain was determined. The classification of each category reflects varying levels of PL among children compared to their same-age peers. Children in the Beginning category exhibit low levels of that variable, those in Progressing category perform similarly to their peers, Achieving category meets the minimum recommended standards, and those in Excelling category exceed required proficiency levels.^34^ These interpretive labels are used only on the tests which adhered directly to the CAPL-2 guidelines (Affective and Cognitive Domains only).

### 2.3 Assessment of the modified physical competence scores

A total of 420 mother-child dyads provided consent for their child to attend a separate physical competence assessment session, with 360 11-year-old children completing a modified test battery of fundamental movement skills (FMS) and physical fitness assessments (Supplementary Table 1). In brief, children completed a modified Körperkoordinationstest Für Kinder (KTK-3) and Faber's Eye-Hand Coordination tests assessed locomotive and coordination skills,^38^^,^^39^ which covered several FMS,^40^

i. walk backwards on 3 balance beams of different widths (6 cm, 4.5 cm, 3 cm);
ii. stand on one platform and hold the other platform, relocate oneself sideways by displacing and picking up the other platform;
iii. jump sideways over a wooden lath; and
iv. execute an eye-hand coordination test by continuously throwing a tennis ball at a target on the wall and catching it with the alternate hand without dropping the ball.

Additionally, children's physical fitness was evaluated using tests of:

i. Handgrip Strength;
ii. Countermovement Jump;
iii. 20-m Sprint;
iv. 5-0-5 Agility; and
v. YMCA Step Test.

These physical fitness tests assessed muscular power, strength, speed, agility, and cardiorespiratory fitness,^41^, ^42^, ^43^, ^44^ each component characterizing one's physical fitness.^45^ The Total Score of Athleticism (TSA), which utilized the sum of z-scores from individual physical test components, was used to form an aggregated score.^46^^,^^47^ This TSA was normalized and scored out of 30 points, consistent with the CAPL-2-inspired weighting system, to derive a novel, non-validated composite score, with proportions categorized into modified category bins (Categories 1-4) based on the modified scoring framework. The score is not directly comparable to the standard CAPL-2 Physical Competency score, as no cross-validation evidence was established for the modified test battery. (See discussion section for more details on generalizability and interpretation of the current study, and Supplementary Table 1 for fitness test scoring details).

### 2.4 Assessment of the modified Overall Physical Literacy scores

The scores from all PL domains were combined and normalized to determine an aggregated score on a 100-point scale representing each child's modified Overall PL, calculated only for children with complete data collected across all three domains (*n* = 360). CAPL-2 age- and sex-specific scores were used as an approximate contextual reference framework for assignment to category bins in order to provide a generalized snapshot of Singapore children's physical literacy status. The aggregated score provided a holistic summary of overall PL status among Singapore children, consistent with the study's aim of characterising PL as a multi-domain construct, and contextualized findings within an established reference framework. Since it was not possible to quantify the Daily Behaviour domain in the present work, the proportional contribution of each domain to the total score differs from the original CAPL-2 weighting structure. The resulting modified Overall PL score therefore cannot be directly compared to a CAPL-2 score; the modified score reported represents a generalized, non-validated composite indicator rather than a true CAPL-2 score.

### 2.5 Assessment of physical activity behavior

PA behavior was assessed via an interviewer-administered questionnaire queried directly to the 11-year-old children. The children recalled their frequency and time spent in MVPA, defined as activities which made them breathe harder or faster than normal, across a typical week. The week's total duration, inclusive of both weekdays and weekends, was averaged to estimate a mean daily MVPA duration. The questionnaire was adapted from the validated Pre-PAQ^48^ to reflect the Singapore context and has been used consistently across multiple GUSTO cohort follow-up visits to maintain continuity with longitudinal surveillance efforts in this population.^49^^,^^50^

### 2.6 Statistical analyses

To characterize PL status in Singapore children, descriptive statistics and frequency distribution of PL category assignments were examined. Chi-square tests of independence were conducted to evaluate differences in distribution of sub-categorical and modified Overall PL scores across sexes and across ethnicity groups. Between-ethnicity comparisons for sub-categorical and modified Overall PL scores were analyzed separately for boys and girls when differences were found between sexes. A Pearson's product-moment correlation was used to assess sub-categorical and modified Overall PL scores against the average self-reported time spent in MVPA, with findings also informing covariate selection for the subsequent regression model. Linear regression determined associations between SES, categorized by household income (S$0-S$1999; S$2000-S$5999; and >S$6000) and highest maternal education level attained (Primary/Secondary; Post-secondary; and University and above), and modified Overall PL scores. The model was further adjusted for sex, ethnicity, and time spent in MVPA, when they demonstrated association with modified Overall PL in prior analyses. Assumptions of normality of residuals, homoscedasticity, linearity, and multicollinearity were verified prior to regression analysis. Chi-square tests of independence, correlation, and regression analyses conducted examined associations between PL outcomes and individual- and environmental-level factors.

Chi-square analyses were also conducted to compare the distribution of sex, ethnicity, and SES between children who completed the modified physical competence assessment (*n* = 360) and those who did not (*n* = 366) for assessment of potential selection bias.

Statistical analyses were performed using jamovi Version 2.3. Statistical significance was set at *p* < 0.05 for all analyses.

## 3 Results

Data from 726 participants (11.0 ± 0.2 years) were analyzed for the affective and cognitive domains through the CAPL-2 questionnaire. Of these, 360 participants completed the questionnaire on PA behavior and the modified physical fitness test battery. Comparisons between completers and non-completers revealed no difference in sex distribution (χ^2^(1) = 0.1, *p* = 0.71). A greater proportion of the completers were, however, of Chinese ethnicity (χ^2^(2) = 13.2, *p* = 0.001), from higher household income group (χ^2^(2) = 17.8, *p* < 0.001), and higher maternal education group (χ^2^(2) = 25.6, *p* < 0.001). Findings from the modified physical competence and Overall PL scores should be interpreted with this caveat in mind. Sociodemographic characteristics are reported in Table 1.

**Table 1:** Sociodemographic characteristics of participants.

Characteristics	n (%)
**Affective and Cognitive Domains**	
*Sex*	
Male	373 (51.4)
Female	353 (48.6)
*Ethnicity*	
Chinese	427 (58.8)
Malay	186 (25.6)
Indian	108 (14.9)
Missing	5 (0.6)
*Household Income*	
S$0-S$1999	99 (13.6)
S$2000-S$5999	369 (50.8)
>S$6000	197 (27.2)
Missing	61 (8.4)
*Maternal Education*	
Primary/Secondary	206 (28.4)
Post-secondary	256 (35.3)
University and above	243 (33.5)
Missing	21 (2.9)
**Physical Competence Domain and Overall Physical Literacy**	
*Sex*	
Male	187 (51.9)
Female	173 (48.1)
*Ethnicity*	
Chinese	235 (65.3)
Malay	80 (22.2)
Indian	42 (11.7)
Missing	3 (0.8)
*Household Income*	
S$0-S$1999	41 (11.4)
S$2000-S$5999	165 (45.8)
>S$6000	122 (33.9)
Missing	32 (8.9)
*Maternal Education*	
Primary/Secondary	72 (20.0)
Post-secondary	134 (37.2)
University and above	143 (39.7)
Missing	11 (3.1)

### 3.1 Physical literacy domains

**Fig. 1:**
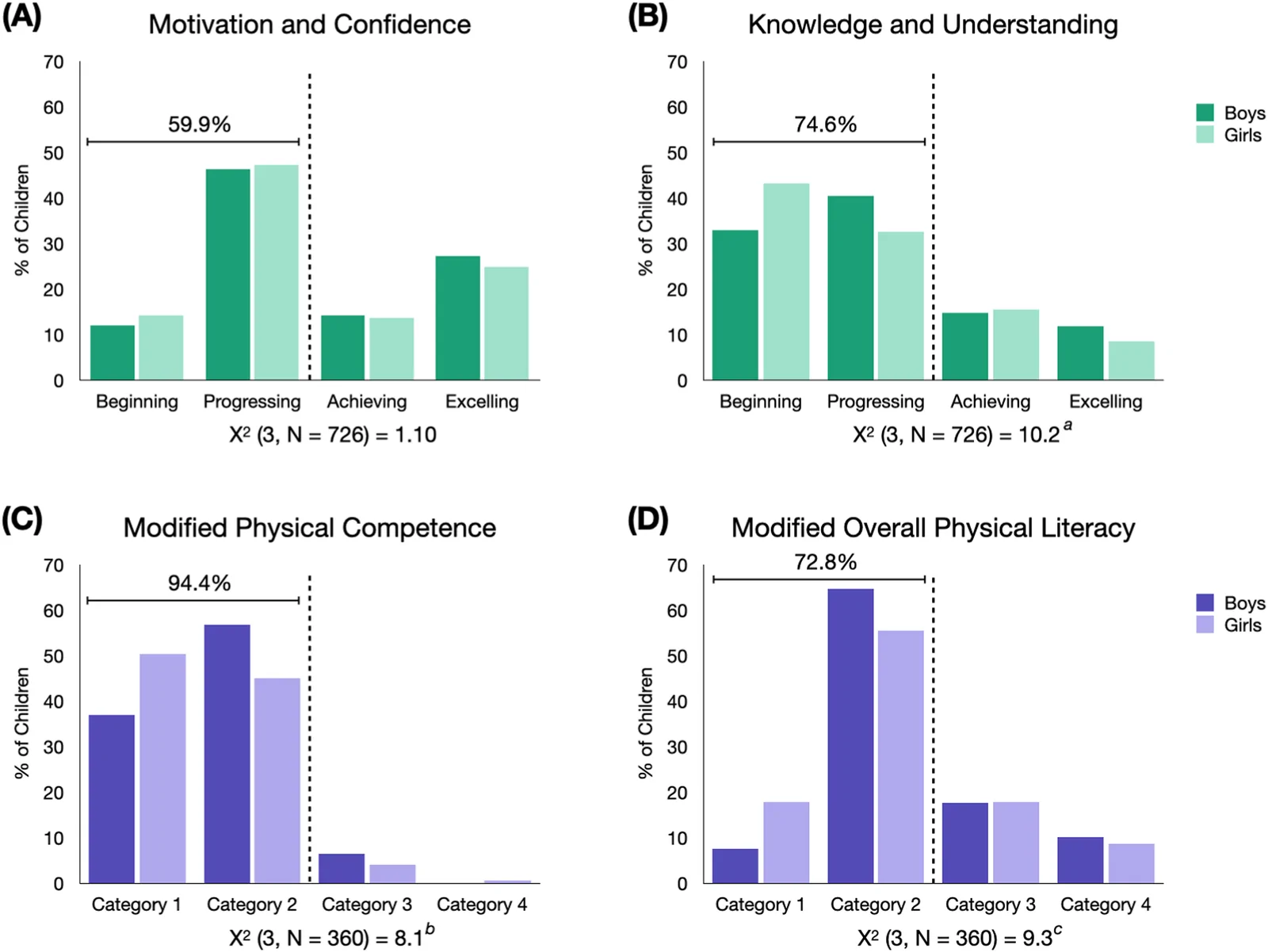
Sub-categorical scores by interpretation category for: (A) Motivation and Confidence; (B) Knowledge and Understanding. Data for modified indicators of (C) Physical Competence; and (D) Overall Physical Literacy, are denoted with categorical data (1-4). All data are stratified by sex. Dotted vertical line indicates cut-off point where minimum health benefits are expected for categories on the right-side. *Note.*^*a*^ Significant association between sex and interpretation category for Knowledge and Understanding domain at *p* = 0.023; ^*b*^ Significant association between sex and interpretation category for the modified Physical Competence domain at *p* = 0.04; ^*c*^ Significant association between sex and interpretation category for the modified Overall Physical Literacy at *p* = 0.03.

In the Motivation and Confidence domain, the mean score was 22.0 ± 4.7, with 59.9% of children (*n* = 726) classified under Beginning and Progressing categories. There were no differences between sex and interpretive categories, (*n* = 726, *p* = 0.78, Fig. 1). For the Knowledge and Understanding domain, the mean score was 5.8 ± 2.3, with 74.6% of the participants (*n* = 726) classified in Beginning and Progressing categories. A greater proportion of girls than boys were categorized in Beginning category (*p* = 0.02, Fig. 1).

For the modified Physical Competence results, the mean score was 14.6 ± 3.2, with 94.4% of participants (*n* = 360) categorized in the first two category bins of the modified scoring framework. A greater proportion of girls than boys were categorized in the first category (*p* = 0.04, Fig. 1). Performance scores for individual fitness tests to assess Physical Competence were compared alongside international normative reference values. Where normative data were available, mean scores derived from this study (for both boys and girls) were below the 50th centile values published from normative benchmarks (Supplementary Table 2).

In all, the mean score was 62.0 ± 9.7, with 72.8% of all children (*n* = 360) were classified in lower bins based on their modified Overall PL score. A greater proportion of girls than boys were assigned to the first category (*p* = 0.03, Fig. 1).

### 3.2 Ethnicity comparisons

**Fig. 2:**
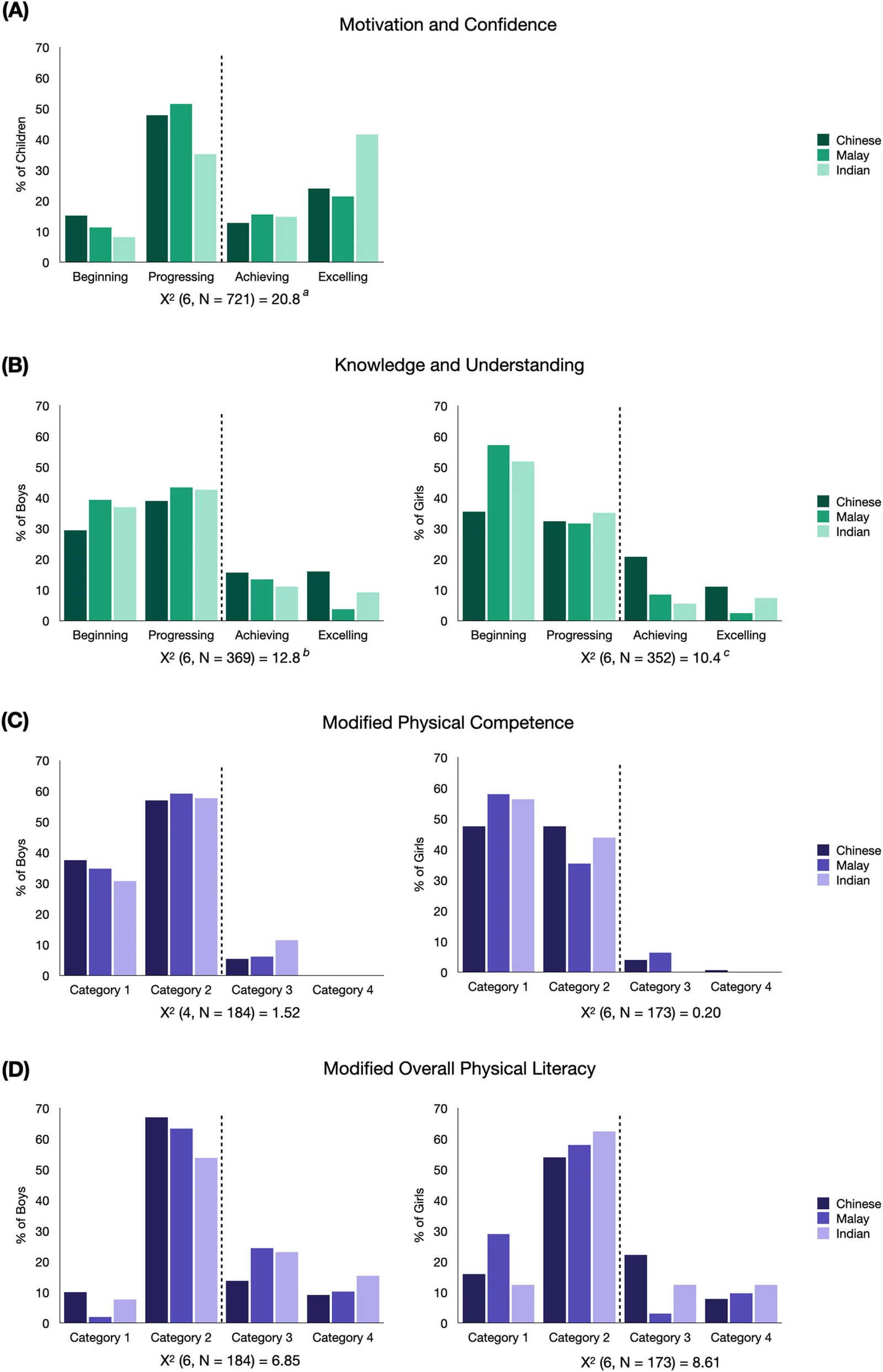
Sub-categorical scores by interpretation category for: (A) Motivation and Confidence; (B) Knowledge and Understanding. Data for modified indicators of (C) Physical Competence; and (D) Overall Physical Literacy, are denoted with categorical data (1-4). Data are stratified by ethnicity for (A) all children combined; and (B-D) boys and girls separately. Dotted vertical line indicates cut-off point where minimum health benefits are expected for categories on the right-side. *Note.*^*a*^ Significant association between ethnicity and interpretation category for Motivation and Confidence domain at *p* < 0.01. ^*b*^ Significant association between ethnicity and interpretation category for Knowledge and Understanding domain in boys at *p* = 0.047; ^*c*^ Significant association between ethnicity and interpretation category for Knowledge and Understanding domain in girls at *p* < 0.001.

Differences were observed between ethnic groups for the Motivation and Confidence domain, (*p* < 0.01, Fig. 2), with a greater proportion of children of Indian ethnicity assigned to Excelling category (17.6% and 20.2% more than Chinese and Malay children, respectively). In the Knowledge and Understanding domain, a greater proportion of Chinese boys were assigned to Excelling category (16.1%) as compared to Malay (3.8%) or Indian ethnicities (9.3%, *p* = 0.047). Similarly, a greater proportion of Chinese girls were assigned to Achieving and Excelling categories (31.9%) compared to Malay (10.9%) or Indian (13.0%) ethnicities, (*p* < 0.001, Fig. 2). There were no differences in ethnicity distribution for the modified Physical Competence results (boys: *p* = 0.82; girls: *p* = 0.82), or the modified Overall PL (boys: *p* = 0.34; girls: *p* = 0.20) (Fig. 2), when categorizing their results using the CAPL-2-inspired scoring framework.

### 3.3 Physical literacy and socio-economic status

**Fig. 3:**
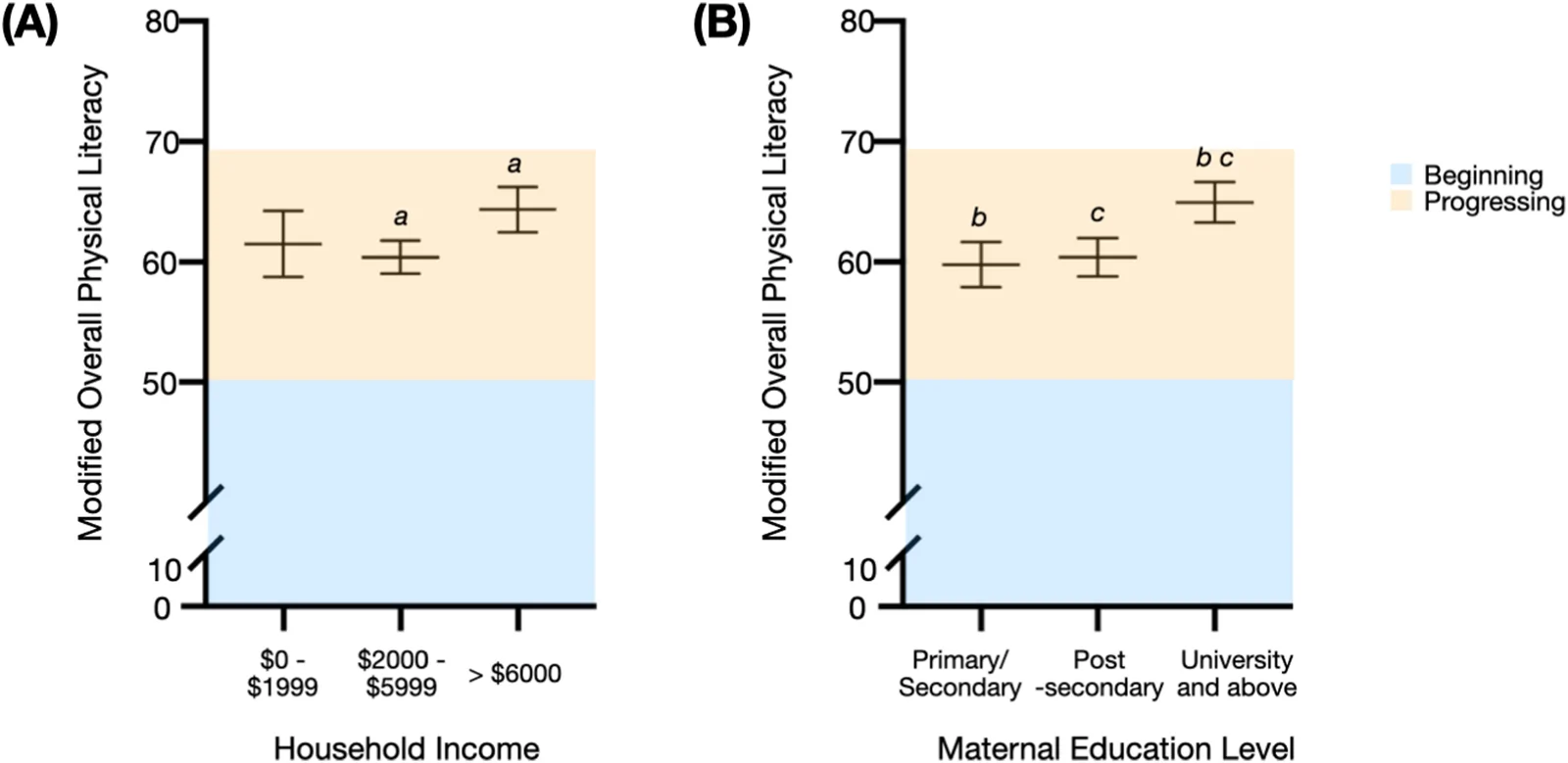
Modified Overall Physical Literacy scores across different (A) Household Income and (B) Maternal Education Level. *Note.*^*a*^ Significant interaction with estimate of 4.04 points at *p* < 0.001; ^*b*^ Significant interaction with estimate of 5.37 points at p < 0.001. ^*c*^ Significant interaction with estimate of 4.55 points at *p* < 0.001. Interpretation categorical scores for Beginning and Progressing are derived from the average sex-specific score for 11-year old boys and girls based on the Canadian Assessment of Physical Literacy (Second Edition) Manual.

Children from the highest household income group displayed greater modified Overall PL scores compared to the middle group, by ∼4.04 points (*p* < 0.001; Fig. 3), based on the 100-point score scale. Similarly, children from the highest maternal education group demonstrated higher scores than the lowest and middle groups by 5.37 and 4.55 points, respectively (*p* < 0.001, Fig. 3). These differences in household income or maternal education classifications were, however, not large enough to affect the modified Overall PL categorizations/bins (i.e. any slight statistical differences observed were not high enough to affect overall distribution of the data).

### 3.4 Associations between physical literacy, physical activity, and socioeconomic status

Individual bivariate correlations comparing mean self-reported time spent in MVPA and individual components of PL were conducted. Positive correlations were observed between the Motivation and Confidence score (r(358) = 0.23, *p* < 0.001), modified Physical Competence score (r(358) = 0.13, *p* = 0.01), and modified Overall PL score (r(358) = 0.23, *p* = 0.01). There was no correlation between time spent in MVPA and the Knowledge and Understanding score (r(358) = 0.07, *p* = 0.13).

Linear regression analyses, adjusted for sex, ethnicity, and time spent in MVPA, found a positive association with the modified Overall PL scores; each additional hour of MVPA was associated with a 2.68 (*p* < 0.001) point increase in the modified Overall PL score, respectively (Table 2). When adjusted for these covariates, differences between household income SES groups disappeared (Table 2). There were slight point differences observed between the lowest (*p* = 0.004) and middle maternal educational attainment groups when compared to the highest maternal education attainment group [4.72 (95% CI 1.48 to 7.96) and 3.98 (95% CI 1.38 to 6.57), *p* = 0.003, Table 2].

**Table 2:** Multiple linear regression analysis of the predictors associated with the Overall Physical Literacy score.

	95% Confidence Interval			
Predictor	Estimate	Lower	Upper	p
**Intercept**	64.07	61.67	66.47	< 0.001
**Socio-economic status by household income (ref: > $6000)**				
$0 - $1999	−0.26	−4.01	3.49	0.892
$2000 - $5999	−2.30	−4.78	0.19	0.070
**Socio-economic status by maternal education level (ref: University and above)**				
Primary/secondary	−4.72	−7.96	−1.48	0.004
Post-secondary	−3.98	−6.57	−1.38	0.003
**Sex (ref: Male)**				
Female	−2.65	−4.65	−0.65	0.010
**Ethnicity (ref: Chinese)**				
Malay	2.32	−0.36	4.99	0.089
Indian	−1.43	−4.64	1.77	0.380
**Time spent in MVPA**	2.68	1.43	3.92	< 0.001

Abbreviation: MVPA = moderate-to-vigorous physical activity.

## 4 Discussion

To address the principle aims of this study, we found that Singapore children exhibit low PL levels, in line with other Asian populations, such that close to 2 in 3 Singapore children have low confidence and motivation in PA participation, and almost 3 in 4 children demonstrate insufficient understanding of the importance of leading a physically active lifestyle to make informed decisions on their behavior choices. More than half of all children measured exhibited low proficiency in physical movement capabilities, including low levels of physical fitness determinants. The majority of children did not reach the 50th centile of international fitness norms for most physical fitness tests assessed in this study. Finally, self-reported MVPA levels were associated with the modified Overall PL index we found in this study.

### 4.1 Physical literacy scores in Singapore children

Low PL levels in this study were broadly consistent with findings communicated from other Asian populations. As a regional example, a study profiling PL levels of children from China and Hong Kong using the CAPL-2 protocol reported that a large proportion of children had low PL.^36^ Across PL domains, the current study found the greatest proportion of children who are inadequately competent in their movement skills, compared to other domains. This finding aligns with observations of low FMS proficiency among 6- to 9-year-old Singaporean children,^51^ and is consistent with the positive association we found between physical competence and time spent in MVPA in the current study. Given the role motor competence, particularly FMS, plays in influencing health-related fitness and sustained PA in children,^52^ low physical competence presents a meaningful opportunity for improvement in Singapore's child population.

The Knowledge and Understanding domain score was not associated with self-reported time spent in MVPA. A discrepancy between self-reported time spent in MVPA and accelerometer-measured PA were also reported in a previous study from this region,^53^ which suggests that children might be overestimating their PA engagement, reinforcing the concept that children in Singapore have limited knowledge on the concept of engaging in, or what constitutes, meaningful PA. A greater proportion of children in this study were found to have limited Knowledge and Understanding compared to the proportion of children who displayed the Motivation and Confidence to partake in PA. This observation is consistent with studies conducted on Chinese and Greek children, where researchers reported that disjointed domains could reinforce a lack of connection between motivation and learning; while children may enjoy partaking in PA, they may have minimal ‘takeaway’ in terms of their cognitive knowledge and/or learning about the greater importance of PA as a whole.^36^^,^^37^

Collectively, these findings are best interpreted within Singapore's unique contextual setting. Academic pursuits and high-stakes examination culture have been shown to displace discretionary time which could be used for PA; parental priorities and time constraints have been identified as key inhibitors to children's PA participation in PA activities outside of school hours.^54^, ^55^, ^56^, ^57^ These barriers can limit children's opportunities to engage in the PA necessary for producing adequate PL development. Singapore's school curriculum mandates 2 to 2.5 h of PE per week,^58^ lower than the WHO's PA guidelines of attaining a weekly average of 60 min/day of MVPA. PE comprises one avenue for attaining adequate quality PA and motor competence development, especially when PE classes are taught by qualified experts,^59^ and particularly for overweight children.^60^ Furthermore, Singapore's densely urbanized built environment may further constrain incidental movement opportunities, as evidenced by comparisons between the physical activity patterns in Thai children from rural school settings, who demonstrated better physical competence than their urban counterparts, with differences attributed to more facilitative environments to provide natural movement.^22^ It should however be noted that PA measures in this study did not distinguish between school-based and community-based activities. Thus, while these factors may influence PA and PL outcomes in children, the direct contribution of school curriculum factors, including PE, cannot be directly determined from this current study.

### 4.2 Individual-level factors affecting physical literacy in Singapore

PL in the current study differed by individual-level factors, such as sex and ethnicity. Consistent within the literature, boys demonstrated higher levels of perceived physical literacy, including perceived (and actual) motor competence, which could be due to differential sport and activity offerings available to boys versus girls (e.g., ball-handling and object-control activities for boys, body-control and rhythmic activities for girls). Different activities on offer may inadvertently foster divergent skill development trajectories and self-perceptions from an early age between girls and boys.^61^ These deviations could have subsequent ‘knock-on’ influences on PA participation and PL development across childhood and motor skills development. There are several sociocultural cues and conventions in Asia, such as aesthetic considerations and lower performance expectations in sports, which could influence both girls' and boys' attitudes and engagement in PA,^62^^,^^63^ deterring girls from being adequately physically literate by the age of measurement in the current work.

Since the majority of Singapore schools are of mixed ethnicity, yet follow a standardized national PE curriculum, out-of-school cultural influences rather than within-school-based exposures are more likely to account for observed ethnicity-related differences. This study found children of Indian ethnicity to be more motivated and confident in PA participation. Parental involvement and role-modelling are critical pathways in which children can develop motivation and confidence in PA.^64^ Cultural factors specific to South Asian communities, including more physically-active parents and the strong influence of sporting celebrities in the media from these nations can facilitate greater PA involvement among Indian youth, for example.^62^^,^^65^ In Singapore's context, a greater proportion of parents or caregivers of children of Indian ethnicity (53.0%) reported being more physically active with, or in front of, their children compared to those of Malay (29.0%) and Chinese ethnicities (22.0%).^66^ These findings are consistent with GUSTO's proxy report where children at 8 years old indicate that 9% and 4% more Indian children are sufficiently active compared to their Chinese and Malay peers, respectively.^66^ Conversely, a greater proportion of children of Chinese ethnicity were in the Achieving and Excelling categories for the Knowledge and Understanding domain. Considering that Chinese mothers' views and behaviors have a lasting and profound effect on their children's lifestyle,^57^ the lower rates of parental modelling (22.0%)^66^ could suggest that knowledge on the importance of PA alone is insufficient to translate to actual time spent in PA, though this hypothesis was not tested in the current study.

### 4.3 Environmental-level factors affecting physical literacy in Singapore

While higher SES is generally associated with more PA participation due to easier access to facilities and opportunities for PA,^67^ the weak and attenuated association between SES and PL found in this study may suggest that the availability of good infrastructure and access to resources does not translate into positive PA behaviors in Singapore. Consistent with Singapore's Active Healthy Kids Report Card, which assigned an A+ for the Community and Environment Indicator, (a score benchmarked by access to facilities, programs, and assurances parents have for PA engagement by children in neighborhood environments),^66^ Singapore received C- grades for the Overall PA and Active Play indicators. A similar discrepancy between excellent availability of physical activity infrastructure, yet poor overall physical activity rates was also observed in culturally comparable cities, like Hong Kong.^68^

The persistence of maternal education as a predictor after model adjustment, unlike household income, may reflect the greater influence of parental education on children's health behaviors in addition to material access, such as health literacy and attitudes towards PA.^69^ However, it should be stated that accessibility to resources was not directly measured within the context of this study, and this interpretation is one aspect of the novel results from the present study, which require further investigation. Other complex environmental, Singapore-level factors may also contribute to the consistently low PL observed across all children regardless of sex, ethnicity, or SES. For instance, higher ambient temperatures beyond 25°C have been associated with reduced time spent in MVPA,^70^ and Singapore's tropical climate may similarly deter spontaneous outdoor PA engagement, which could influence PL development.^71^ However, since climatic data were not collected within the scope of this study, future research into the PA habits of children growing up in hot, tropical climates should be investigated.

### 4.4 Implications for future policy development, educational practice, and public health intervention

The association between shorter PA duration and lower Overall PL via our modified index suggests the need for targeted, multi-level interventions in Singapore. Given that children spend a considerable proportion of their time in school, school-based programs represent an effective approach to improving PA behaviors, particularly through their sustained implementation of multicomponent interventions like PA breaks and extracurricular programmes.^72^ Outside of school, public health campaigns targeting parents and caregivers are needed to shift cultural priorities towards valuing PA alongside other academic achievements, in recognition that physically literate, active children are more likely to achieve better (e.g. academic, physical and mental health) outcomes across the lifespan. Advocating for more systematic physical fitness testing, perhaps using simplified, shorter test batteries (e.g. YFIT) which adhere to international expert consensus,^73^ may help introduce this concept of value, and contribute to greater knowledge and understanding on the many lifelong health benefits provided.

### 4.5 Study considerations

This study represents the first attempt to characterize PL in Singapore children using a multi-component assessment tool, contributing to regional knowledge and providing benchmark data for future surveillance efforts. The multi-ethnic representation of the cohort, Singapore's cultural similarities to East and Southeast Asian population, and its ecological validity, are valuable additions to the paucity of child physical literacy data currently available in the region. Although the CAPL-2 instrument was not directly validated in Singapore's highly specific, multi-ethnic population, it has been implemented in Asian and Southeast Asian contexts before, including preliminary validations in Indonesian schoolchildren.^21^^,^^36^

This study is not without its limitations. Cross-sectional assessment of PL provides a temporal snapshot which may not fully encapsulate the fluid nature of PL. Data collection was conducted during the COVID-19 pandemic restriction period, which had several direct, negative effects on PL assessment capabilities, specifically: (i) We did not use pedometers or accelerometers to assess objective assessment of the Daily Behavior domain. Although the self-reported PA portion was administered, this measurement alone does not constitute a complete domain score. (ii) This study used a modified fitness test battery due to space constraints and social distancing requirements mandated during the COVID-19 period, in place of the standard CAPL-2 protocols for assessing the Physical Competency domain. (iii) Physical competence and Overall PL scores derived from, or which include, the modified fitness test battery are not directly comparable to CAPL-2. (iv) There may have been a perceived ‘invasiveness’ of the full, in-person fitness test battery from a parental perspective, although ‘parental apprehension’ was not measured within the scope of this study. Anecdotal observations during data collection suggested that reluctance from some parents contributed to the lower sample size for the physical competency domain only. Furthermore, a greater proportion of children who completed the modified physical competence assessment were of Chinese ethnicity and from higher SES groups, and this subsample may therefore not be fully representative of the broader recruited sample. Findings from the modified physical competence domain and Overall PL score should be interpreted with this consideration.

Collectively, the overall PL score in this study does not constitute a true CAPL-2 score, due to the omission of the Daily Behavior domain and rescaling of the remaining domain scores, which led to an altered proportional contribution of each domain relative to the original CAPL-2 weighting structure. This composite score therefore represents a novel, modified instrument rather than a true CAPL-2 score for the physical competence and overall PL portions of the protocol; comparisons to the established CAPL-2 norms are not directly comparable. The CAPL-2 normative category sorting was retained as a reference framework for contextualising the scores attained, but should be interpreted with caution.

## 5 Conclusion

Universally-low levels of PL observed in Singapore school children warrant concern. Poor PL may extend beyond Singapore, given the similarities in educational systems, cultural influences, and socioeconomic factors present among countries within the region. Increasing PE curriculum time, and supplementing the duration of PA with after-school activities, or shifting the focus in the school curriculum to improve children's knowledge and understanding of the importance of PA as a lifelong preventative health outcome tool, is suggested to better facilitate the development of PL in children within Singapore, especially since higher self-reported MVPA durations were associated with higher PL scores.

## Author's contributions

VLLT was involved in the conceptualization and design of the present study, conducted data collection, data analysis, data visualization, and drafting of the manuscript. SAM was involved in the data visualization, and drafting of the manuscript. YSL, YSC, and FY conceptualized and designed the cohort study. JGE conceptualized and designed the cohort study and the present study. JLKW conceptualized and designed the present study. All authors were involved in the data interpretation, provision of critical feedback during manuscript preparation, and approval of the final version of the manuscript.

## Funding

The study is supported by the National Research Foundation (NRF) under the Open Fund-Large Collaborative Grant (OF-LCG; MOH-000504) administered by the Singapore Ministry of Health's National Medical Research Council (NMRC) and the Agency for Science, Technology and Research (A*STAR). In RIE2025, the study is supported by funding from the NRF's Human Health and Potential (HHP) Domain, under the Human Potential Programme.

## Conflict of interest disclosures

Jason Kai Wei Lee is a member of the Editorial Board of Journal of Exercise Science and Fitness. He was not involved in the journal’s peer review process of, or decisions related to, this manuscript. The other authors have no conflicts of interest relevant to this article.
