# Supplementary material for: Limited physical literacy outcomes in Singapore children: Implications for public health practice
Source: J Exerc Sci Fit. 2026 Sep 2;24(4):200517. doi: 10.1016/j.jesf.2026.200517 (PMC13578625; doi:10.1016/j.jesf.2026.200517)
Supplement: Supplementary file 1 [file mmc1.docx]

**Supplementary Table 1** Comparison of the standard CAPL-2 Physical Competence assessment protocol and the modified fitness test protocol employed in the present study.

| **Component** | **Standard CAPL-2 Protocol** | **Current Study Protocol** | **Current Study Scoring Details** |
| --- | --- | --- | --- |
| Movement Skill | Canadian Agility and Movement Skill Assessment (CAMSA) | Modified KTK-3 (walk backwards, move sideways, jump sideways) and Eye-Hand Coordination test | Walk Backwards (sum of all beams):  Maximum of 8 steps per trial, 3 trials per beam, across 3 beams (maximum: 72 steps).^1^  Move Sideways (sum of 2 trials):  Number of successful sideways relocations completed within 20 seconds per trial.^1^  Jump Sideways (sum of 2 trials):  Number of sideways jumps completed within 15 seconds per trial.^1^  Eye-Hand Coordination (best of 2 trials):  Number of successful alternate-hand catches per trial.^1^ |
| Speed | Assessed as part of the CAMSA  (Not measured independently) | 20m Sprint | Time to complete a 20-m straight-line sprint measured using timing gates; fastest of 2 trials. |
| Agility | Assessed as part of the CAMSA  (Not measured independently) | 5-0-5 Agility | Time to complete a 10-m sprint, 180° change of direction, and 5-m return sprint; fastest of 2 trials. |
| Musculoskeletal performance | Isometric Plank Hold | Handgrip Strength and Countermovement Jump | Maximum force recorded using a handgrip dynamometer; best of 3 trials.  Highest jump height recorded on a force plate, initiated from a stationary jumping position, followed by a preparatory countermovement involving a knee flexion; best of 2 trials. |
| Aerobic Fitness | Progressive Aerobic Cardiovascular Endurance Run (PACER) | YMCA Step Test | Estimated VO₂peak derived from heart rate at pre-exercise, 1-minute, and 2-minute post-exercise recovery following 3 minutes of stepping at 96 BPM. Calculated using the formula: VO₂max = −2.045 + (height in inches × 0.062) + (step test average heart rate as a percentage of resting heart rate × −0.411) + (resting heart rate × 0.011).^2^ |
| Score Derivation | Sum of normalised CAMSA score (10 pts) + PACER (10 pts) + Plank hold (10 pts) = 30 pts total | Total Score of Athleticism (TSA) via sum of z-scores across all test components, normalised to 30 points. |  |

*Note.* Physical competence scores derived from the modified protocol are not directly equivalent to scores derived from the standard CAPL-2 protocol.

Abbreviations: CAPL-2 = Canadian Assessment of Physical Literacy (Second Edition); CAMSA = Canadian Agility and Movement Skill Assessment; PACER = Progressive Aerobic Cardiovascular Endurance Run; KTK = Körperkoordinationstest Für Kinder; BPM = beats per minute; VO₂peak = peak oxygen uptake; TSA = Total Score of Athleticism.
