# Supplementary material for: Limited physical literacy outcomes in Singapore children: Implications for public health practice
Source: J Exerc Sci Fit. 2026 Sep 2;24(4):200517. doi: 10.1016/j.jesf.2026.200517 (PMC13578625; doi:10.1016/j.jesf.2026.200517)
Supplement: Supplementary file 2 [file mmc2.docx]

**Supplementary Table 2** Comparison of Physical Competence test scores in Singapore children (n = 360) against available international normative reference values for 11-year-old children

| **Test** | **Boys** | | **Girls** | | **Reference Population** |
| --- | --- | --- | --- | --- | --- |
|  | **Current Study**  **Mean ± SD** | **Normative Reference**  **50^th^ Percentile** | **Current Study**  **Mean ± SD** | **Normative Reference**  **50^th^ Percentile** |  |
| **Walk Backwards** | 34 ± 13 | 54 | 36 ± 11 | 55 | Coppens et al. (2021)^1^;  Belgian children and adolescents;  n = 208 (boys); 101 (girls) |
| **Move Sideways** | 43 ± 8 | 50 | 40 ± 7 | 51 |  |
| **Jump Sideways** | 58 ± 13 | 73 | 53 ± 13 | 70 |  |
| **Eye-Hand Coordination** | 14 ± 7 | 39 | 12 ± 7 | 21 |  |
| **Handgrip Strength (kg)** | 16.5 ± 3.9 | 19.0 | 16.4 ± 3.7 | 17.5 | Tomkinson et al. (2018)^2^;  European children and adolescents (Eurofit);  n = 11 358 (boys); 11 582 (girls) |
|  |  | 20.2 |  | 20.2 | Zhang et al. (2021)^3^;  Chinese children and adolescents;  n = 3859 (boys); 3428 (girls) |
| **Countermovement Jump (cm)** | 21.8 ± 4.9 | 27.0 | 20.6 ± 4.0 | 22.0 | Ramírez-Vélez et al. (2017)^4^;  Colombian schoolchildren (FUPRECOL);  n = 414 (boys); 619 (girls) |
| **20-m Sprint (s)** | 4.22 ± 0.38 | 4.00 | 4.38 ± 0.39 | 4.17 | Štefan ^1^ et al. (2022)^5^;  Croatian children and adolescents (CROFIT);  n = 910 (boys); 952 (girls) |
| **5-0-5 Agility (s)** | 3.23 ± 0.31 | No age- and sex-matched normative reference available | 3.34 ± 0.30 | No age- and sex-matched normative reference available |  |
| **YMCA Step Test (L/min)** | 2.19 ± 0.29 |  | 2.26 ± 0.24 |  |  |

*Note.* Normative reference values represent the 50th percentile for children aged 11 years from the cited studies. Normative values for the 5-0-5 agility test and YMCA step test are unavailable for paediatric populations and are therefore reported descriptively only. Lower sprint and agility times indicate better performance.

Abbreviations: SD = standard deviation; VO₂peak = peak oxygen uptake.

References:

1.	Štefan L, Neljak B, Petrić V, Kasović M, Vespalec T. Normative data for musculoskeletal fitness in 13,217 children and adolescents: The Croatian Fitness (CROFIT) study. *Research quarterly for exercise and sport*. 2022;93(3):528-536.

2.	Coppens E, Laureys F, Mostaert M, D'Hondt E, Deconinck FJ, Lenoir M. Validation of a motor competence assessment tool for children and adolescents (KTK3+) with normative values for 6-to 19-year-olds. *Frontiers in physiology*. 2021;12:652952.

3.	Tomkinson GR, Carver KD, Atkinson F, et al. European normative values for physical fitness in children and adolescents aged 9–17 years: results from 2 779 165 Eurofit performances representing 30 countries. *British journal of sports medicine*. 2018;52(22):1445-1456.

4.	Zhang F, Bi C, Yin X, et al. Physical fitness reference standards for Chinese children and adolescents. *Scientific reports*. 2021;11(1):4991.

5.	Ramírez-Vélez R, Correa-Bautista JE, Lobelo F, Cadore EL, Alonso-Martinez AM, Izquierdo M. Vertical jump and leg power normative data for colombian schoolchildren aged 9–17.9 years: The FUPRECOL study. *The Journal of Strength & Conditioning Research*. 2017;31(4):990-998.
